# Mitochondrial Metabolism in Myocardial Remodeling and Mechanical Unloading: Implications for Ischemic Heart Disease

**DOI:** 10.3389/fcvm.2021.789267

**Published:** 2021-12-09

**Authors:** Min Jiang, Xiaoye Xie, Feng Cao, Yabin Wang

**Affiliations:** ^1^Department of Cardiology, National Clinical Research Center for Geriatric Disease, The Second Medical Center, Chinese People's Liberation Army General Hospital, Beijing, China; ^2^College of Pulmonary and Critical Care Medicine, Chinese People's Liberation Army General Hospital, Beijing, China; ^3^Medical School of Chinese People's Liberation Army, Chinese People's Liberation Army General Hospital, Beijing, China; ^4^Department of Cadre Ward, The 960 Hospital of Chinese People's Liberation Army, Jinan, China

**Keywords:** ischemic heart disease, heart failure, mitochondrial metabolism, veno-arterial ECMO, mechanical unloading, left ventricular assist device

## Abstract

Ischemic heart disease refers to myocardial degeneration, necrosis, and fibrosis caused by coronary artery disease. It can lead to severe left ventricular dysfunction (LVEF ≤ 35–40%) and is a major cause of heart failure (HF). In each contraction, myocardium is subjected to a variety of mechanical forces, such as stretch, afterload, and shear stress, and these mechanical stresses are clinically associated with myocardial remodeling and, eventually, cardiac outcomes. Mitochondria produce 90% of ATP in the heart and participate in metabolic pathways that regulate the balance of glucose and fatty acid oxidative phosphorylation. However, altered energetics and metabolic reprogramming are proved to aggravate HF development and progression by disturbing substrate utilization. This review briefly summarizes the current insights into the adaptations of cardiomyocytes to mechanical stimuli and underlying mechanisms in ischemic heart disease, with focusing on mitochondrial metabolism. We also discuss how mechanical circulatory support (MCS) alters myocardial energy metabolism and affects the detrimental metabolic adaptations of the dysfunctional myocardium.

## Introduction

As estimated by World Health Organization (WHO), ischemic heart disease (IHD) is the leading cause of death worldwide (1). IHD stands for disease syndromes caused by myocardial ischemia due to disproportionate supply and demand of oxygenated blood perfusion in the heart (2). The insufficient oxygen, decreased nutrient levels, as well as deficient substrates and metabolites, are all pathophysiological contributors for IHD. Myocardial ischemia is usually induced by diminished coronary blood flow due to atherosclerosis-induced coronary artery occlusion, clinically manifesting as coronary heart disease or coronary artery disease.

IHD affects cardiac metabolism and function in different stages, ranging from angina to acute myocardial infarction (AMI) and HF. In myocardium, mitochondria produce 90% of ATP, making mitochondrial oxidative phosphorylation (OXPHOS) the primary energy source for the heart. Besides, mitochondria also participate in metabolic pathways that regulate the balance of glucose and fatty acid oxidative phosphorylation. Under physiological conditions, mitochondria keep a dynamic and homeostatic state in regulating metabolic pathways and cellular ions, which lay foundation for normal cellular functioning. Any deviation from this homeostatic state will lead to mitochondrial dysfunction, including elevated mitochondrial Ca^2+^, damage of mitochondria membranes and enzymes and defects of electron transport chain (ETC), all of which are contributing factors for cellular apoptosis and necrosis (3).

In terms of interventions, options for IHD mainly focus on reestablishing the oxygen supply-demand imbalance, which is pivotal for clinical prognosis for patients with IHD. Recently, although related approaches have advanced by leaps and bounds, there are still certain numbers of patients who are refractory to conventional treatment, and some of them develop heart failure (HF) after IHD interventions. As heart is a metabolically active organ, metabolism modulation is promising in the treatment of IHD as energy metabolism is closely related with disease progression.

## Mitochondrial Metabolism in Normal Heart

As a circulatory pump, heart requires cardiomyocytes to generate enormous quantities of ATP to sustain the uninterrupted and orderly coordinated systolic and diastolic function. The main fuels of the myocardium are fatty acids (FAs), glucose, pyruvate, lactate, ketone, and amino acids, which are acquired from the blood continuously for the cardiomyocytes cannot store these energy substrates. As previously reported, the majority of energy (60–90%) required for myocardial activity is derived from free FAs, and the remaining 10–40% originates from carbohydrates and amino acids (4). The myocardial energy metabolism mainly includes substrate utilization, oxidative phosphorylation, and ATP transfer and utilization. Firstly, acetyl-CoA derived from β-oxidation of FAs and glycolysis is synthesized in mitochondria and enters the tricarboxylic acid (TCA) cycle. Under various physiological processes, the healthy heart is metabolically flexible in shifting between substrates to maintain energy production. Then, oxidative phosphorylation is completed in the mitochondria through the respiratory chain and generates the direct energy source ATP. Finally, through the creatine phosphate energy shuttle, creatine kinase (CK) in the mitochondria catalyzes ATP and creatine and generates phosphocreatine (PCr) and adenosine diphosphate (ADP). The generated PCr immediately transfers from mitochondria to myofibrils, where it interacts with ADP and synthesizes ATP and creatine under the catalyzation of myocardial CK so that cardiomyocytes can use ATP for contraction and relaxation and maintain the energy-consuming activity of the heart.

## Mitochondrial Metabolism in IHD

The energy metabolism dysfunction caused by IHD includes alterations of substrate utilization, dysfunction of OXPHOS, impairment of ATP synthesis, and mitochondrial dysfunction. Under physiological conditions, fatty acid oxidation (FAO) generates more ATP than glucose oxidation per molecule oxidized at the expense of greater O_2_ consumption. In IHD and HF, the O_2_ supply to the myocardium is insufficient due to reduced tissue perfusion. Accordingly, both FAO and carbohydrate oxidation decline, and production of ATP is disrupted. To compensate the reduced O_2_ shortage, the ATP production in the myocardium switches from aerobic to anaerobic mode, and glycolysis becomes a more predominant source of energy as it yields 11–13% more ATP per unit of consumed O_2_ than β-oxidation (5). Besides, under ischemic and hypoxic conditions, large quantities of activated long-chain FAs produced by fat mobilization accumulate in cells and mitochondrial membranes due to decreased or stopped β-oxidation of FAs. These long-chain FAs can result in cardiomyocyte damage and death, and suppress the activity of pyruvate dehydrogenase (PDH), thereby limiting the aerobic oxidation of glucose, reducing the ATP production efficiency, increasing the content of intracellular lactate and hydrogen ions, and, finally, disrupting the myocardial contractility (3, 6).

### Oxygen Consumption

It has been increasingly apparent that ischemia and reperfusion (IR)-induced alterations in the kinetics of O_2_ supply-to-demand equilibrium depend seriously on the duration and severity of IR. The effects of ischemia exist from short, beneficial ischemia (<10 min) to moderate, reversible ischemia (10–20 min) to severe, irreversible ischemia (>20 min). In reversible ischemia, recovery of overall oxidative metabolism recovers much slower to a preischemic level and couples to contractile function (7). However, in irreversible ischemia, the recovery of oxygen consumption and metabolism is fast, and the mechanical function is severely damaged (8). The IR-induced increases in Ca^2+^ partially explain the rapid recovery of metabolism at early reperfusion in irreversible ischemia, whereas the increased glycolytic rate from exogenous glucose induced by IR results in slower recovery of O_2_ consumption-to-demand balance at late reperfusion (9). The concentration of free Ca^2+^ is implicated in regulating several intramitochondrial enzymes, including the PDH, the isocitrate dehydrogenase, and the oxoglutarate dehydrogenase, thus leading to elevated carbohydrate oxidation during early reperfusion (10). This supports the notion that the mitochondrion is the first organelle to display signs of injury under irreversible ischemia, and that regulating mitochondrial energetics within the cell is an early protective strategy of reversible ischemia (9). Moreover, the rapid metabolic recovery uncoupled from mechanical function is a surrogate for poor prognosis of IR injury. Therefore, interventions aiming at attenuating reperfusion blood flow or inhibiting mitochondrial function are considered beneficial (11, 12). In the reperfusion stage following ischemia, FAO generates ATP as a primary energy source at the cost of glucose oxidation negatively impacts the cardiac function in the isolated hearts and in patients with coronary artery disease (6, 13).

### Glucose Metabolism

**Figure 1:**
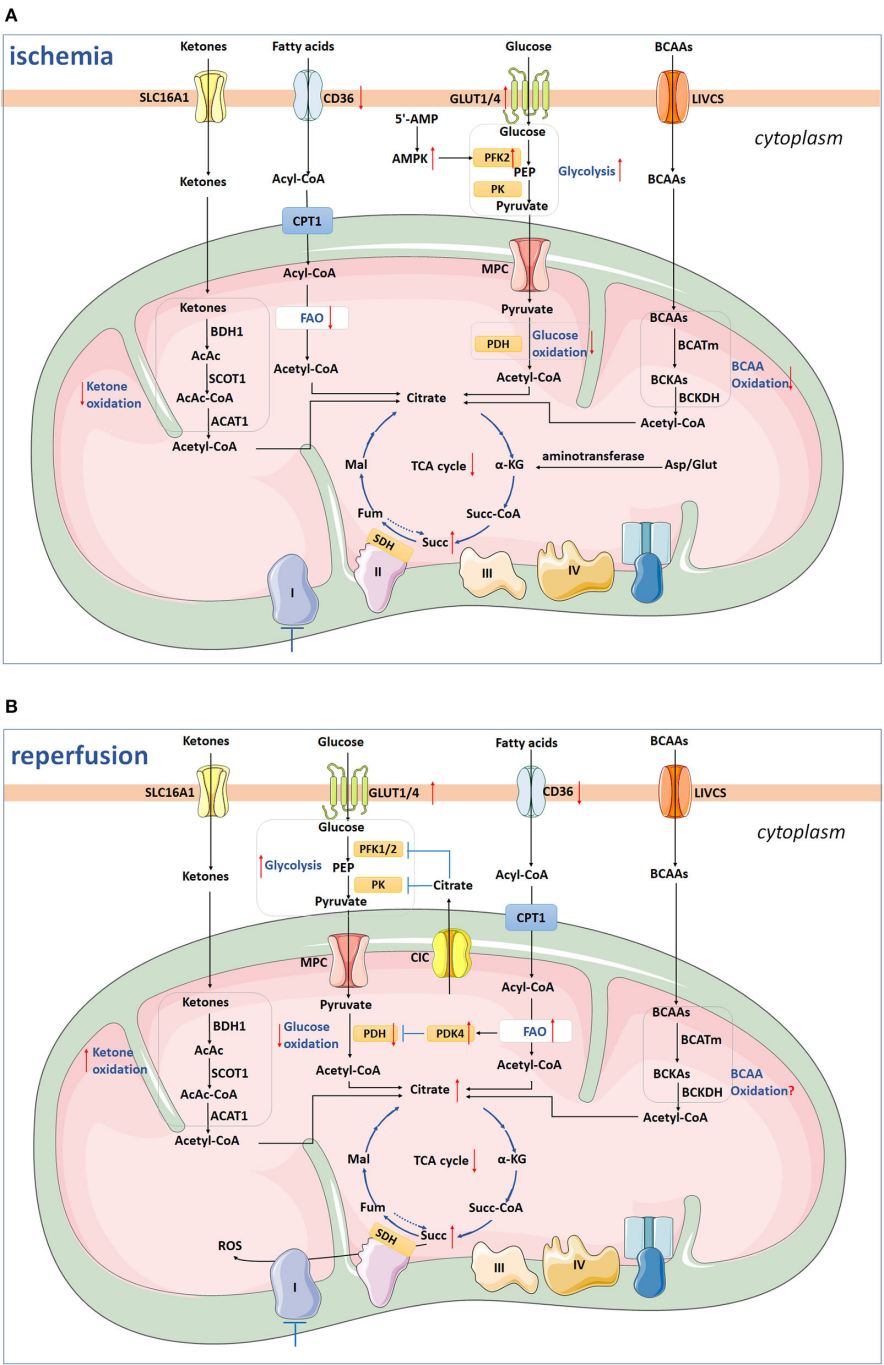
The scheme depicts ketone oxidation, glycolysis, glucose oxidation, fatty acid oxidation, BCAA oxidation, and succinate metabolism under ischemia **(A)** and reperfusion **(B)**. **(A)** Under ischemia, GLUT4-mediated glucose uptake and glycolysis are increased by AMPK-induced PFK-2 activation, whereas glucose oxidation is shuttled down. The CD36 on sarcolemma, FAs uptake, and FAO are inhibited. Besides, BCAAs and ketones oxidation are also inhibited during ischemia. The TCA cycle and Complex I are inhibited, while the succinate is accumulated from existing metabolites of TCA with mitochondrial Complex II reversal and aminotransferase anaplerosis. **(B)** During reperfusion, CD36 remains low, while FAO returns to the pre-ischemic level. High levels of NADH, acetyl-CoA, and ATP in mitochondria generated from increased FAO inhibit activated PDH *via* activating PDK4 and, therefore, inhibit glycolysis and pyruvate oxidation. The glycolysis still remains high, while glucose oxidation is inhibited. Ketones utilization is increased, while the level of BCAAs oxidation remains to be clarified in the future. Two-thirds of succinate enters into perfusate, and the remaining one-third of succinate is oxidized *via* SDH, driving ROS burst. SLC16A1, Solute carrier (SLC) 16A1/monocarboxylate transporter 1 (MCT1); β-OHB, β-hydroxybutyrate; AcAc, acetoacetate; BDH1, β-hydroxybutyrate dehydrogenase 1; SCOT, succinyl-CoA:3 oxoacid-CoA transferase; AcAc-CoA, catalyze acetoacetyl-CoA; ACAT1, acetyl-CoA acetyltransferase 1; CPT1, carnitine palmitoyl transferase 1; FAO, fatty acids oxidation; GLUT1/4, glucose transporter 1/4; PEP, phosphoenolpyruvate; PK, pyruvate kinase; MPC, mitochondrial pyruvate carrier; PDH, pyruvate dehydrogenase; BCAAs, branched chain amino acids; LIVCS, Leu, Ile, Val: cation symporter; BCKAs, α-keto-acids; BCATm, mitochondrial branched chain aminotransaminase; BCKDH, branched chain α-keto acid dehydrogenase; α-KG, α-ketoglutarate; Fum, fumarate; Mal, malate; Succ, succinate; Succ-CoA, succinyl-CoA; SDH, succinate dehydrogenase; Asp, aspartate; Glut, glutamine. The arrow facing up represents an increase, and down indicates a decrease. Blue lines with a T shape represent inhibition.

Glucose metabolism mainly includes anaerobic glycolysis, aerobic oxidation, pentose phosphate pathway (PPP), uronic acid pathway, glycogen synthesis and decomposition, gluconeogenesis, and hexose metabolism, etc. As mentioned above, under hypoxia and ischemia, the aerobic oxidation of FAs is diminished and glycolysis increases and glucose becomes the main energy substrate for myocardial energy metabolism. The uptake of glucose depends on glucose transporters (GLUTs) on sarcolemma of cardiomyocytes, and GLUT1 and GLUT4 are two major isoforms in cardiomyocytes. GLUT1 dominates during fetal and early postnatal life, whereas GLUT4 is the main isoform in adult cardiomyocytes. GLUT4 has a higher affinity for glucose than GLUT1, which makes GLUT4 functioning as the major glucose transporter in adult heart (14). Upon ischemia and hypoxia, GLUT4 translocates to cell membrane from the intracellular vesicles and then augments glucose intake (Figure 1A), exhibiting a protective role during cardiac ischemia in neonatal or adult mouse (15–17). Besides, GLUT4 depletion represses glucose use during ischemia and facilitates progression of irreversible cardiac dysfunction associated with blunted ATP generation during IR injury (18). After being transported into cytosol, glucose is phosphorylated by hexokinase, generating glucose 6-phosphate (G6P). Then, G6P is converted into pyruvate by glycolytic enzymes, including phosphofructokinase 1 (PFK1), PFK2, and pyruvate kinase (PK), and the pyruvate can either be metabolized to lactate or be transported into the mitochondrial matrix through the mitochondrial pyruvate carrier (MPC) for oxidation. Under ischemia, the accumulation of 5'-AMP leads to activation of AMP-activated protein kinase (AMPK), which phosphorylates and activates phosphofructokinase-2 (PFK-2) to promote glycolysis (19, 20). Recently, elevated MPC1 and MPC2 have been found in the surviving tissue of the heart subjected to IR injury, indicating a compensatory mechanism that enhances mitochondrial pyruvate uptake to limit intracellular acidosis (21). Upon transportation into mitochondria, pyruvate is converted to acetyl-CoA by pyruvate dehydrogenase (PDH) and further metabolized in the TCA cycle. During ischemia, glucose oxidation is completely shuttled down (Figure 1A), and the recovery of it is regulated by FA oxidation as FA competes with glucose for oxidation.

During myocardial reperfusion, the level of O_2_ and substrates are restored, whereas glycolysis still remains high, because lactate and inorganic phosphoric acid are washed away which diminish the inhibition of glycolysis, and dysfunctional mitochondria cannot use O_2_ effectively. However, glucose oxidation is inhibited during the early stage of reperfusion. As mentioned above, AMPK is activated during ischemia and the accumulated AMPK inactivates ACC at the early stage of reperfusion (8). The decreased ACC activity, in combination with malonyl-CoA degradation leads to decreased malonyl-CoA levels during reperfusion, relieving inhibition of CPT1 and, finally, resulting in upregulated FA oxidation (22, 23). Increased FA oxidation generates high levels of acetyl-CoA and NADH/NAD^+^, which inhibits the enzyme activity of PDH through activating PDK4 and therefore inhibits pyruvate oxidation (8). The excessive fatty acids β-oxidation facilitates the accumulation of intermediates from the TCA cycle and efflux of citrate to cytosol *via* the mitochondrial citrate carrier (CIC)/SLC25A1, and high levels of cytosolic citrate can suppress PFK-1/2 and pyruvate kinase (PK) activities, and thus diminish glycolysis, pyruvate generation, and oxidation (24, 25). However, as demonstrated before, the Ca^2+^ overload during reperfusion may conquer this inhibition by activating PDH and then increasing glucose oxidation (10, 26). Above all, glucose oxidation is inhibited at an early stage of reperfusion after moderate ischemia, while may increase following more severe or prolonged ischemia (27, 28).

Data from *in vivo* and *ex vivo* studies suggested that promoting glucose oxidation during perfusion is closely correlated with reduced IR injury and improved cardiac function. In *ex vivo* studies, interventions increasing glucose oxidation target the PDH flux, including boosting PDH activity with dichloroacetate, supplementing pyruvate or facilitating glucose uptake by applying high glucose/insulin or overexpressing GLUT1 transporter (29–31). Simultaneously, repressing FAs uptake or FAO also relieves inhibition of glucose oxidation (32, 33). As the improved function observed in the short reperfusion period mostly indicates the accelerated recovery of mechanical function and enhanced re-energization, the long-term post-ischemic myocardial salvage is to be assessed. Therefore, *in vivo* studies administrating glucose oxidation-stimulating substances, such as dichloroacetate, phosphonate compounds, or high-density lipoproteins (HDL) prior to reperfusion reduced infarct size several days after reperfusion (34–36).

Although increased glycolysis only marginally increases ATP production, it promotes flux into the metabolic pathways branching from glycolysis, such as polyol and hexosamine biosynthetic pathways that could independently activate signaling pathways responsible for myocardial remodeling (37, 38). Besides, it has been suggested that glycolysis is intrinsically linked to the redox state of cell (39). Glucose metabolized through glycolysis and the branching PPP in the cytosol feeds the TCA cycle in the mitochondria and produces major reductant e.g., NADH, NADPH for thiol-dependent antioxidant defense. Activation of PPP results in upregulated NADPH/NADP^+^ and GSH/GSSG couples and an improved redox state, which relieved acute IR injury in cultured cardiomyocytes and mouse hearts (40).

### Fatty Acid Oxidation

After uptake into cardiomyocytes, FAs are esterified to fatty acyl-CoA in cytosol and then transferred to carnitines by carnitine palmitoyl transferase 1 (CPT1), which localizes in the inner surface of mitochondrial outer membrane (41). The acylcarnitines are transferred into mitochondrial matrix by carnitine acylcarnitine translocase (CAT) and then converted back into fatty acyl-CoA by CPT2. The fatty acyl-CoA is catalyzed to acetyl-CoA for β-oxidation in mitochondria.

Fatty acids metabolism dysfunction includes FAs and carnitine transport disorder, CoA dehydrogenase deficiency, impairment of enzymes, ketones production disorder, etc. CPT-1 is the rate-limiting enzyme for β-oxidation and is inhibited by Malonyl-CoA, which is synthesized by acetyl-CoA carboxylase (ACC) from acetyl-CoA using biotin and ATP as cofactors. The level of malonyl-CoA in the heart is regulated both by its synthesis and degradation, and AMPK, ACC, and malonyl-CoA decarboxylase (MCD) are extremely important. AMPK phosphorylates and inhibits ACC, which reduces the production of malonyl-CoA and phosphorylates as well as activates MCD, promoting the reverse reaction and converting malonyl-CoA into acetyl-CoA (42). As demonstrated before, AMPK was rapidly activated as an energy sensor to regulate FFAs uptake and oxidation under pathophysiological conditions of hypoxia and ischemia. In cardiomyocytes, AMPK promotes long-chain-FAs uptake through FA translocase (CD36) (43). AMPK activation and relative increase of MCD decrease myocardial malonyl CoA content, which then alleviates inhibition of CPT1 and subsequently facilitates FAO as increased fatty acyl CoA moieties enters into the mitochondrial matrix (44). During mild ischemia, this facilitates fatty acid β-oxidation to be the primary and continuous contributor for oxidative ATP generation, which leads to a decreased level of glucose oxidation, despite increased glycolysis (45). Besides, lactate production by LDH from pyruvate and an increased level of NAD^+^ from NADH are also promoted in order to sustain anaerobic glycolysis. The uncoupling of glycolysis and glucose oxidation leads to an accumulation of lactate and H^+^, aggravating the ionic disturbance induced by ischemia. However, it should be noted that the accumulated lactate and H^+^ are immediately eliminated from the cardiomyocyte under conditions of hypoxia and mild ischemia.

With total ischemia, the FAO is inhibited due to the accumulated reducing equivalents and disturbance of acyl CoA dehydrogenase and 3-hydroxyacyl CoA dehydrogenase enzyme reactions (Figure 1A). CD36 on sarcolemma is downregulated by 32%, and the FAO rate decreases by 95% (46). The inhibition of fatty acid β-oxidation inevitably leads to accumulation of metabolic intermediates in various cell compartments. Fatty acyl carnitines accumulate in cytosol and the mitochondrial matrix, while fatty acyl-CoA mainly deposits in mitochondrial matrix because the CoA pool does not exchange with the small cytoplasmic CoA pool (47). The excessive amounts of acylcarnitine accelerate ROS production and increase the probability of mitochondrial permeability transition pore (mPTP) opening, and excessive cytosolic acyl-CoA causes loss of ΔΨm from outside of mitochondria (47, 48). This inhibits the formation of amorphous intramitochondrial densities and disrupts mitochondrial cristae, both of which may eventually induce mitochondrial dysfunction (49). Therefore, the approaches aiming at regulating FA metabolism for cardioprotection in IR injury should first evaluate the potential impact on overall energy homeostasis and possible cardiotoxicity carefully. Recently, studies have shown that inhibition of FAO reduces damage induced by IR using inhibitors, which limit FA transport and metabolism pathways (32, 50–54).

In the stage of perfusion, the activation of AMPK and increased MCD activity still persist, leading to marked decrease of malonyl CoA content. Therefore, the rates of FAO rapidly return to its pre-ischemic level during reperfusion at the expense of glucose oxidation with the CD36 level remained low (Figure 1B) (46), whereas the myocardial contraction is still depressed. However, this causes the uncoupling of glucose metabolism and thus deteriorates intracellular acidosis, which increases the risk of cardiac arrhythmias and decreases the contractility because the response between contractile proteins and Ca^2+^ is repressed (55). Therefore, the myocardial recovery is hindered despite the restoration of coronary perfusion.

### Ketone Body Oxidation

Ketone bodies [acetoacetate, acetone, and β-hydroxybutyrate (β-OHB)] are synthesized in the liver using acetyl CoA that predominately derived from FAO. They are increasingly being recognized as an important energy substrate of the myocardium, especially in HF (38, 56). After entering mitochondria, they rapidly form acetyl-CoA *via* β-OHB dehydrogenase (BDH1), succinyl-CoA:3-oxoacid-CoA transferase (SCOT) and mitochondrial acetyl-CoA acetyltransferase 1 (ACAT1) (38). The predominant ketone body oxidized in heart is β-OHB. In terms of O_2_ consumption for ATP generation, ketones are much more efficient than FAs but less efficient than glucose (4).

Increasing ketone oxidation rates in failing hearts increases overall energy production without compromising glucose or fatty acid metabolism, albeit without increasing cardiac efficiency (57). Previous studies showed that short-term fasting reduces infarct size and limits the duration of ventricular tachycardia occurring at early reperfusion, and this is correlated with increased concentration of β-OHB and β-OHB/acetoacetate ratio (58). However, whether ketonemia induced by fasting in mice after IR injury protect or deteriorates myocardial injury is conflicting (59, 60). Furthermore, *in vivo* studies suggested that the effect of ketonemia may be boosted by exogenous ketone therapy: fasting with ketone infusion led to the least myocardial damage compared with fasting without ketone infusion or a fed state with ketone infusion in rat hearts after coronary occlusion (61). As β-OHB conferred substantial protection against oxidative stress in multiple organs (62, 63), further mechanism study also confirmed that reduced mitochondrial ROS, enhanced ATP generation, attenuated mitochondrial swelling, and partly restored mPTP in myocardium account for the cardio protection against IR injury (64). To determine ketones utilization under ischemic condition in humans, the concentrations of coronary and systemic serum lactate and β-OHB were measured in 171 consecutive patients with angina (65). The results showed that increased lactate production was associated with decreased myocardial β-OHB consumption, demonstrating suppressed utilization of ketone bodies under ischemia in the coronary circulation (Figure 1A) (65).

HF is the next stage of myocardial infarction. A mouse model of progressive HF induced by transverse aortic constriction (TAC)/myocardial infarction exhibited upregulated BDH1 along with upregulated genes encoding ketone body transporters (MCT1 and MCT2) and increased 13C-BHB use on the basis of nuclear magnetic resonance studies (66). Cardiac-specific BDH1 knockout mice exhibit more severe ventricular remodeling and dysfunction after TAC/myocardial infarction (66), whereas BDH1 overexpression attenuates cardiac remodeling and DNA damage in a pressure-overloaded model (67). Increased availability of 3-OHB significantly reduced ventricular remodeling and improved mitochondrial energetics caused by TAC/myocardial infarction in wild-type mice compared with mice-fed normal chow (68). Except for pathway manipulation and ketone supplementation, the cardioprotective effects of SGLT2 inhibitors on infarcted hearts can also be attributed to increased myocardial ketone utilization (69, 70).

### BCAA Metabolism

Branched chain amino acids oxidation is also an origin of ATP generation by the heart (71). In mitochondria, leucine, isoleucine, and valine are firstly transaminated to branched chain α-keto-acids (BCKAs) by the mitochondrial branched chain aminotransaminase (BCATm), and then decarboxylated to form CoA compounds by the mitochondrial branched chain α-keto acid dehydrogenase (BCKDH). The products of BCKDH will then generate acetyl-CoA, which fuels for the TCA cycle.

High levels of circulating BCAA and its derivative BCKAs are correlated with increased incidence of cardiovascular disease (72, 73). Previous studies revealed that plasma and cardiac levels of BCAA and BCKAs increase in the rodent model of myocardial IR injury (74, 75). Impaired cardiac BCAA oxidation exacerbates cardiac insulin resistance and directly contributes to cardiac dysfunction and remodeling post AMI (74, 76), while BCAA supplementation aggravates cardiac dysfunction and increases infarct size following coronary artery ligation-induced myocardial infarction (74). As BCAA oxidation rates in heart only supply 1–2% of the total cardiac ATP (75), it is impossible that interventions increasing BCAA catabolism itself have substantial impact on cardiac ATP generation. Recently, BCAA and their intermediates have been recognized to affect glucose and FA metabolism (76–78). In the heart, chronic accumulation of BCAA inhibits PDH activity through suppressing protein O-linked-N-acetylglucosamine (O-GlcNAc) modification, leading to significantly reduced glucose oxidation, and, therefore, rendering the heart vulnerable to IR injury (76). Dietary or genetic factors-induced BCAA accumulation enhances cardiac FAO levels *via* upregulating PPAR-α expression transcriptionally, thereby triggering lipid peroxidation toxicity and rendering the heart vulnerable to IR injury (78). As BCKAs have been shown to trigger ROS formation (79), promoting oxidative decarboxylation of BCKAs through PPC2m overexpression reduces the oxidative stress and eventually alleviates myocardial IR injury (80). However, in contrast with this, another study found that BCKAs attenuate acute IR injury in hearts by inhibiting oxidative stress-induced cell death and protecting mitochondria and energy production (81). The discrepancy may be attributed to the manner of BCKAs exposure and the severity of injury/stress. Moreover, it has been proved that blockade of mitochondria ETC at different complexes inhibits cardiac IR injury (23, 82). BCKAs repress activity of complex I in cardiac mitochondria (83), which capacitate its function as mitochondrial targets for cardioprotection.

### Succinate Metabolism

When exposed to ischemia, succinate, a metabolite of the TCA cycle, accumulates and is oxidized by succinate dehydrogenase (SDH) which drives enormous ROS production by mitochondrial complex I (84). Previous study by Zhang and colleagues found that succinate mainly originates from TCA cycle using existing metabolites, such as fumarate, malate, oxaloacetate, citrate, and aconitate, with only trivial contributions from mitochondrial complex II reversal and aminotransferase anaplerosis during heart ischemia (Figure 1A) (85). Glucose derived from glycogen also seems to play a part in ischemia induced-succinate accumulation without the requirement of mitochondrial pyruvate entry, suggesting that glucose is not the main source of carbohydrates (85). The α-ketoglutarate (α-KG)-derived succinate is protective at the first few minutes of ischemia as it contains GTP generation through phosphorylating succinyl-CoA synthetase at the substrate level, which defers ischemia-induced ATP exhaustion in cardiomyocytes (86).

Not all succinate that accumulates during ischemia is oxidized by SDH, with approximately two-thirds of them release into the perfusate *via* MCT1 under low pH conditions during early reperfusion (87). The remaining one-third succinate are quickly oxidized within a few minutes of reperfusion, which diminishes the CoQ pool and also hyperpolarizes the inner membrane of mitochondria (84). Together, these alterations drive reverse electron transport (RET) at complex I, which produces ROS dramatically (Figure 1B) (84, 88). Besides, succinate oxidation induces generation and secretion of pro-inflammatory factors, such as IL-1β, and simultaneously inhibits production of anti-inflammatory factors (89).

Clinically, the succinate concentration in arterial, coronary sinus, and peripheral venous blood of patients with STEMI is higher than that in patients with non-STEMI or stable angina, and the release of cardiac succinate in STEMI is correlated with the severity of acute myocardial injury (90). The emerging therapeutic and cardioprotective strategy targeting succinate metabolism *via* SDH inhibition includes preventing its accumulation during ischemia (less oxidizable succinate during reperfusion) and directly depressing its oxidation during reperfusion (91). The TCA cycle intermediate malate and the succinate competitor malonate are known SDH inhibitors (86). SDH inhibition with malonate before ischemia or at the onset of reperfusion reduces infarct size in isolated mice hearts through inhibiting ROS generation and mPTP opening (84, 92). The discovery that one third of accumulated succinate during ischemia is oxidized within the first 5 min of reperfusion (and 59% of which is within the 1st min) suggests that early reperfusion is a critical time window for therapeutic targeting of complex II (85). Furthermore, SDH inhibition by malonate administration at 1-week post-MI facilitates adult cardiomyocyte proliferation, revascularization of the infarct zone, and myocardial regeneration following infarction (93). Above all, succinate represents a promising biomarker for evaluating severity of AMI, and inhibition of SDH proposes a key metabolic target to render protection following infarction.

### Epigenetic Aspects of Metabolic Signaling

Most of the previously described substrate utilization and the metabolic process are a result of gene transcription. During the last decades, epigenetics, including DNA modification and posttranslational modification of proteins, are increasingly studied to delineate how metabolites regulate gene expression. Lysine acetylation, one of the posttranslational modifications, can regulate many non-histone proteins, which are involved in gene transcription, mRNA splicing, signal transduction, cell metabolism and survival (94). Sirtuins (SIRT1-7) are Class III histone deacetylases (HDACs), which participate in IR injury *via* regulating mitochondrial metabolism. In the heart mitochondria of SIRT5 knockout mice, mass spectrometry suggests that lysine succinylation is increased and the substrate of SIRT5 includes proteins that participate in FA metabolism, TCA cycle and OXPHOS (95). Compared with wild-type littermates, SIRT5 knockout exacerbates infarction, and this is reversed by pretreatment of malonate, implicating SIRT5 modulates protein succinylation in IR injury. SIRT3 is the primary mitochondrial lysine deacetylase; its knockdown significantly aggravates the extent of IR injury compared to wild-type hearts (96). The enhanced mitochondrial protein acetylation, which is closely correlated with the inhibited activity of complex I and MnSOD, is the underlying mechanism. In the sepsis model, depletion of SIRT3 also induces excessive acetylation of enzymes in the TCA cycle and production of lactate and NADH, which accordingly deteriorate sepsis-induced cardiac dysfunction (97). However, recently, a dual knockout mouse model targeting carnitine acetyltransferase and SIRT3 resulted in a dramatically proteome-wide upregulation of lysine acetylation in heart mitochondria, which exhibited a ~86% overlap with characteristics of HF, while this has no or minimal impact on mitochondrial function, such as dehydrogenase activity, respiration, redox state and OXPHOS (98). Therefore, clinical and mechanistic studies are to be done to determine protein acetylation in mitochondrial metabolism and its actual contribution to IR injury.

Except for acetylation and succinylation, new acylation mediated by metabolites themselves is founded to participate in epigenetic regulation of gene expression and enzymatic activity. In an HFpEF (HF with preserved ejection fraction) mice model, by partly activating citrate synthase through lysine β-hydroxybutyrylation and inhibiting FA uptake, β-OHB limits the acetyl-CoA pool and thus inhibits mitochondrial acetylation (99). Besides, the crotonylation of histone also regulates gene transcription and metabolism. In hypertrophic cardiomyopathy, Enoyl Coenzyme A Hydratase1 (ECHS1), the enzyme in the second step of fatty acids β-oxidation, reduces the intracellular crotonyl-CoA level and inhibits cardiomyocytes hypertrophy *via* repressing H3K18cr and H2BK12cr and NFATc3 expression (100). Recently, Zhang et al. have discovered that lactate, the product of glycolysis, can stimulate histone lactylation, which participates in M1 macrophage polarization by inducing *Arg1* expression (101). The histone lactylation is later found to regulate tissue repair and cancer progression (101–103). As macrophage phenotype and inflammation are closely correlated with prognosis of IHD and HF (104, 105), the role of this “lactate clock” in cardiovascular system is to be clarified to develop novel therapeutics.

## Mechanical Circulatory Support on Myocardial Mitochondrial Metabolism

In spite of timely perfusion, over 1/4 of patients suffering the first-time AMI develop HF within 1 year (106). For every 5% increase in the myocardial infarct size, the 1-year HF hospitalization and 1-year mortality will increase by 20% (107). The reason for the poor prognosis of patients with AMI is that reperfusion paradoxically worsens myocardial injury. Upon reperfusion, the ischemic myocardium is subjected to abrupt biochemical and metabolic alterations, including mitochondrial re-energization, ROS burst, intracellular and mitochondrial Ca^2+^ overload, rapid pH correction and inflammation (108). These alterations can lead necrosis of endothelial cells and cardiomyocytes *via* opening of mPTP and hypercontraction. The mPTP is a non-selective channel on inner membrane of mitochondria, and its opening disrupts the mitochondrial membrane potential, which uncouples OXPHOS, exhausts ATP, and induces cell death. During ischemia, mPTP is closed, while the reperfusion-induced ROS burst, Ca^2+^ overload, and rapid pH fluctuation cause mPTP opening (109). Therefore, mitochondria, acting as a signaling hub, are an important target for cardioprotection. Improved and more efficient strategies are in urgent demand to limit myocardial injury by inhibiting deleterious response and inducing a cardioprotective mechanism prior to reperfusion of the infarct-related arteries so as to prevent the development of patients with HF after MI. Recently, increasing numbers of mechanical circulatory support (MCS) devices have been applied in clinical practice as they can enhance systemic mean arterial pressure and concurrently reduce left ventricular (LV) wall stress and stroke work, which may be beneficial for myocardial oxygen supply-demand equilibrium (110–112).

### Mechanical Loading on Mitochondrial Metabolism of Myocardium

Normally, cardiomyocytes are subjected to three types of mechanical loading, including cyclic stretch imposed by every heartbeat, shear stress generated by flowing blood, and static stretching due to blood pressure. Cardiomyocytes are sensitive to mechanical stress, which are transduced to molecular signaling by biomechanical sensors. The sensors and transduction pathways have been studied for several years in the hope of preventing development and progression of cardiovascular diseases by shutting down mechanotransduction.

Emerging evidence has shown that excessive mechanical loading can disturb mitochondrial homeostasis, including dynamics, metabolism, Ca^2+^ homeostasis and redox state (113, 114). Ca^2+^ leak is the earliest alteration observed under mechanical loading, and during myocardial systole, the duration and amount of Ca^2+^ leak determine the contractile force of myocardium (115). Ca^2+^ is a versatile signaling molecule, which regulates numerous cellular processes, including cell metabolism, and the mitochondrial Ca^2+^ homeostasis is determine by equilibrium between Ca^2+^ influx and efflux, which are regulated by Ca^2+^ channels, transporters, exchangers and subcellular organelles (116, 117). Ca^2+^ affects metabolic processes such as the TCA cycle through allosteric regulation of enzymes or indirectly *via* regulating phosphatases and kinases (118, 119). In addition, Ca^2+^ channels, transporters and exchangers are also reported to be responsible for metabolism (120–122). The Ca^2+^ release-activated Ca^2+^ (CRAC) channel, which imports Ca^2+^ from endoplasmic reticulum and extracellular space, is involved in FA metabolism (119). Piezo1, which is discovered in 2010 by Coste et al. and forms cationic non-specific channels, also participates in mechanotransduction by transducing ionic current and Ca^2+^ influx (123). In cardiovascular system, Piezo1 expresses in endothelial cells, smooth muscle cells, cardiomyocytes, and cardiac fibroblasts and can be activated by cyclic stretch and shear stress. In endothelial cells, Piezo1 couples shear stress-provoked Ca^2+^ entry and endothelial cell organization and alignment *via* Ca^2+^-activated proteolytic enzyme calpain (124). However, data on the presence and function of Piezo1 in cardiomyocytes are limited. Liang et al. found that the mRNA and the protein level of Piezo1 were upregulated in HF after myocardial infarction (125). In another *in vitro* model, Piezo1 was reported to participate in sensing cyclic stretch and can activate the mechanical low-density lipoprotein (LDL) receptor-related protein 6 (LRP6)/β-catenin signaling pathway (126). Clinically, the increased Piezo1 mRNA level was observed on monocytes of patients with aortic valve stenosis, which increases shear stress on circulating blood cells, and transcatheter aortic valve implantation (TAVI) significantly decreased the mRNA level on monocytes (127). Further *in vitro* mechanistic study suggests that Piezo1-mediated Ca^2+^ influx from intracellular stores, as well as the extracellular environment, was involved in monocyte activation. Apart from Ca^2+^ homeostasis, Piezo1 constitutes a regulator of the Hippo-Yes-associated protein (YAP)/PDZ-binding motif (TAZ) pathway (128, 129), which is involved in converting mechanical stress into biochemical signal and has been well-studied (130). With regular mechanical stress, the activated Hippo proteins phosphorylate YAP/TAZ and thus confine them to cytoplasm. Upon excessive mechanical loading, Hippo is inhibited and YAP/TAZ enters into nucleus where it interacts with the TEAD1 family to induce gene transcription. YAP and TAZ can regulate metabolism of various substrates and mitochondrial function, which has been reviewed in detail previously (131). However, the direct evidence that Piezo1 regulates mitochondrial metabolism is lacking.

### LV Unloading by LV-To-Aorta Circulatory Support

Over the past decades, preclinical studies have demonstrated that, compared with reperfusion alone, mechanical unloading of the left ventricle prior to coronary reperfusion can reduce IR injury and myocardial infarct size in AMI (132, 133). Latterly, preclinical studies also indicated that LV unloading and delayed reperfusion by 30 min can initiate cardioprotective changes, including upregulated SDF-1a/CXCR4 expression, increased cardioprotective signaling, and reduced apoptosis (133, 134). Compared with reperfusion alone, 30 min of LV mechanical unloading before reperfusion was adequate to diminish LV infarct size, improve cardiac function, and downregulate biomarkers related to undesirable remodeling and HF 28 days post AMI, thus promoting recovery of myocardial function 30 days after AMI (135). Furthermore, mechanical unloading increased collateral coronary flow and myocardial microvascular perfusion by reducing the LV-end diastolic wall stress (136, 137). In order to determine the safety and feasibility of LV unloading and delayed reperfusion of patients with STEMI, the DTU-STEMI pilot trial (Door-To-Unload in STEMI Pilot Trial) randomly assigns 50 patients with anterior STEMI to LV unloading with immediate reperfusion or LV unloading followed by 30-min delay before reperfusion (138). The results of the trial suggested that, despite delayed reperfusion, LV unloading does not increase the infarct size, which lays a good foundation for a future pivotal trial. In addition, in patients with large anterior STEMIs, which is defined as a sum of precordial ST-segment elevation STE ≥ 6 mm, LV unloading reduces infarct size normalized to the area at risk compared with reperfusion alone (138). In patients with LAD occlusion and STE ≥ 7 mm, LV unloading also diminishes the infarct size quantified by cardiac magnetic resonance at 3- to 5-day post-MI compared with that in the perfusion-alone group (139).

### LV Unloading on Myocardial Mitochondrial Metabolism

**Figure 2:**
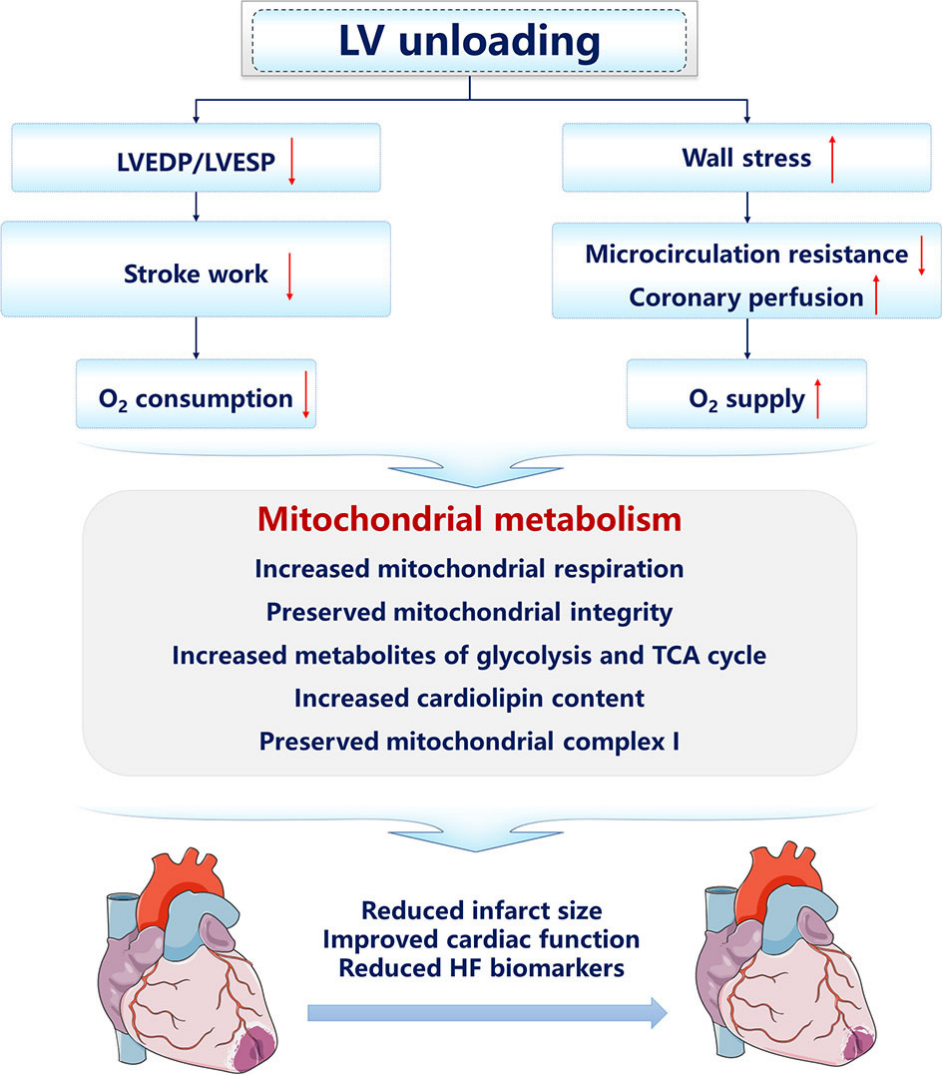
The cardioprotective mechanisms of left ventricular unloading. The arrow facing up represents an increase, and down indicates a decrease.

Recently, studies focusing on mitochondrial integrity and function under LV unloading have been increasing. The improvement of mitochondrial dysfunction is firstly reported in patients with HF supported with long-term left ventricular assist device (LVAD) therapy (140). To elucidate the mechanisms of the clinical benefit brought by LVAD, tissue specimens were harvested from the explanted hearts at the time of transplantation or LVAD implantation. Mechanical unloading supported by LVAD increased the respiratory rate and efficiency of mitochondrial respiration on TCA cycle-derived substrates in isolated mitochondria, suggesting that mitochondrial metabolism is involved in the cardioprotective effect of LVAD (140). In failing human myocardium, decreased oxidation and increased accumulation of long-chain FAs in the hypertrophic heart are accompanied by increased acyl-derived intermediates, including lipotoxic ceramides that curtail cardiac function. Mechanical unloading *via* LVAD implantation increased the content of acyl CoA, facilitating the lipid trafficking and amelioration of the lipotoxicity (141–143). Besides, by decreasing LV afterload, LVAD reduces acyl chains required for energy production of the myocardium, which, therefore, restores the upstream acyl CoA pool (143). In the IR injury model, compared with pure reperfusion, LV unloading for 30 min prior to reperfusion upregulates genes associated with mitochondrial function and respiration, and maintains the integrity of mitochondria in the infarct zone (135). And tissue samples from the infarct zone with or without LV unloading following perfusion were analyzed using untargeted metabolomics. Compared with the sham control group, IR significantly reduces the levels of key metabolites related to glycolysis, glucose oxidation, fatty acid transport and oxidation, and amino acid use, while LV unloading preserves the levels of these metabolites (139). Pathway analysis also showed that, compared with reperfusion alone, LV unloading remarkably altered various pathways involved in glycolysis, the TCA cycle, and ETC function, suggesting that LV unloading preserves substrate levels for OXPHOS (139). Further functional studies showed that, compared with IR, LV unloading retains intact mitochondrial structure, including cardiolipin content and activity of ETC, including mitochondrial Complex I, and reduces oxidative stress in mitochondria isolated from the infarct zone. In conclusion, by reestablishing the oxygen supply-consumption balance, the primary clinical prognosis determinant of patients with IHD, LV mechanical unloading preserves myocardial energetics and mitochondrial function, and, therefore, limits the infarct size and improves cardiac function after AMI (Figure 2). Nevertheless, more preclinical and human studies concerning LV mechanical unloading require to be done since the panorama of substrate utilization and mitochondrial metabolism is incomplete.

### Metabolic Biomarkers of Myocardial Recovery Under LV Unloading

Recently, the effects of mechanical unloading on mitochondrial metabolism in patients with progressive HF, including ischemic and non-ischemic cardiomyopathy, have increasingly investigated to identify whether progression and improvement of HF are correlated with altered myocardial energetics and the metabolic process induced by mechanical unloading. In the ischemic myocardium, altered distribution of CK isoenzyme may disrupt energy transfer between mitochondria and the contractile apparatus. Cardiolipin is the characteristic phospholipid of the mitochondrial inner membrane whose functional status is a determinant for efficient mitochondrial coupling. Previous studies have shown that LVAD support can improve mitochondrial coupling in ischemic cardiomyopathy (ICM) hearts, and the improvement is associated with the normalization of cardiolipin composition (140, 144).

In normal hearts, lactate can be converted to pyruvate by lactate dehydrogenase (LDH) to enter the TCA cycle and generate ATP, and concurrent lactate production balances the lactate consumption (145). During HF, this balance is disturbed, with an increased glycolytic pyruvate-derived lactate and a simultaneous decrease in lactate utilization (146). Diakos et al. demonstrated that glycolytic metabolites are increased in patients with post-LVAD HF without a corresponding increase of early TCA cycle intermediates, which may be attributed to poor recovery of mitochondrial oxidative capacity and volume density post-LVAD (146). Besides, the subsequent intermediates after succinyl CoA anaplerotic access and amino acids levels are increased post LVAD unloading, representing a complementary energy source that could fuel the TCA cycle and building blocks for protein synthesis (146). Especially, they revealed that patients with better myocardial recovery post LVAD seem to have improved mitochondrial function and structure with LVAD unloading. This is verified in subsequent study showing that MPC1 was lower in myocardial tissue samples of patients with HF than those of non-failing donors (147). The MPC transports pyruvate into the mitochondria for the TCA cycle and is an important modulating point, which determines whether pyruvate is oxidized or converted to lactate (148). In these patients with chronic HF, increased MPC1 abundance post-LVAD is observed in responders (patients with significantly improved myocardial function after LVAD unloading) but not non-responders, suggesting defective mitochondrial pyruvate metabolism may be a primary factor contributing to their HF. Further animal models and *in vitro* cardiomyocyte models proved that inhibited mitochondrial pyruvate oxidation is closely correlated with cardiomyocyte hypertrophy.

Except for glucose oxidation, the metabolites of glycolysis may be channeled to accessory pathways of glycolysis. Clinically, LVAD implantation enhances levels of glucose and glucose-6-phosphate in patients with ICM (149). Compared with post-LVAD non-responders, rate-limiting enzymes of PPP and one-carbon metabolism, which protect myocardium through producing NADPH to promote biosynthesis and antagonizing oxidative stress, are significantly increased in post-LVAD responders (150). The responders also have improved mitochondrial density and increased expression of α-dystroglycan that maintains the extracellular matrix and cytoskeletal integrity (150).Therefore, the deleterious mitochondrial or related metabolism pathway may be underlying etiology for the cardiac response to LVAD, and specific metabolites may be utilized as cardiac prognostic biomarkers of LVAD implantation. However, concerning LVAD study on HF that includes patients with ischemic and non-ischemic cardiomyopathy, caution should be made when interpreting these results as studies have shown that metabolic processes can be used to distinguish non-ischemic dilated cardiomyopathy from ICM (151).

### Hemodynamics of VA-ECMO

Cardiac arrest or cardiogenic shock (CS) occurs in ~3–10% of patients with AMI and is associated with about 30–50% in-hospital mortality (152, 153). Therefore, percutaneous circulatory support, particularly venoarterial extracorporeal membrane oxygenation (VA-ECMO), which belongs to RA-To-Aterial circulatory support, is increasingly used to provide hemodynamic support for the management of AMI. Since VA-ECMO transfers blood from venous reservoir to the arterial system, the afterload is increased due to the retrograde flow. Thus, the volume status and native ventricular function of the patients will affect LV volume. In patients with low volume in the venous system, VA-ECMO initiation reduces the total cardiac preload and therefore LV volume (154). However, as VA-ECMO is usually applied to CS patients with venous congestion, the pressurized arterial system will increase systemic blood pressure. When the original LV function is retained, the increase in LV systolic pressure will overcome the LV afterload to enable blood ejection *via* aortic valve, and the increased flow and LV systolic blood pressure will not come at the cost of LV diastolic blood pressure when the LV contractility is preserved (154). In patients with LV dysfunction compromised by extensive anterior wall MI, the diminished stroke volume results in increased LV systolic and diastolic pressures because the dysfunctional LV cannot pump blood effectively to resist increased afterload and the pressurized arterial tree (110). The elevated LV afterload increases wall stress of LV and left atrial and therefore increases oxygen demand, which finally hinders myocardial recovery (155). The extra LV afterload and insufficient LV mechanical unloading under VA-ECMO may lead to severe complications, including LV stasis, thrombosis, pulmonary edema, and ventricular dilatation-induced ischemia, all of which increase mortality (156, 157). Particularly, ECMO can reduce both LV end diastolic volume and LV systolic wall stress of the normal hearts but increase LV wall stress in ischemic hearts (158).

To decompress the LV and attenuate increased afterload *via* elevating forward flow, the VA-ECMO is combined with other mechanical devices in clinical practices (159). In a retrospective cohort from America, almost 60% of AMI admissions had a second temporary MCS device besides ECMO, and 30.3% of them were placed concomitantly (160). Previous meta-analysis suggests that ECMO + IABP strategy achieves lower mortality compared with ECMO alone in CS of AMI (161). Compared with that of patients with VA-ECMO alone, the mortality was lower in patients with combined Impella and VA-ECMO (162, 163). Besides, LV unloading done early (within 12 h) seems to be correlated with an increased rate of successful weaning and decreased short-term mortality (164). Recent studies have suggested that combined utilization of Impella to decompress the LV with ECMO reduced the mortality rate of CS (154). However, as VA-ECMO support systemic perfusion at the risk of increasing LV load, available data on VA-ECMO utilization in AMI are limited, and most of them are retrospective. There is an urgent need to conduct randomized controlled trials (RCT) to investigate the application of VA-ECMO in AMI and its effects on infarct size as well as long-term prognosis.

### VA-ECMO on Mitochondrial Metabolism of Normal Hearts

As recovery of myocardial function is the primary prerequisite for weaning from the ECMO circuit, it is necessary to fully clarify the effects of changing energy equilibrium and substrate utilization of the heart under ECMO support. Of all these factors, hemodynamics of the heart during ECMO cause changes in substrate metabolism. Even if no direct myocardial damage has occurred before, ECMO can cause cardiac arrest. Therefore, the previously uninjured and unenlarged heart is used to define the effect of ECMO on substrate use and myocardial energetics to exclude the interferences of hypoxia, ischemia, or reperfusion. In infants, systemic studies have shown that ECMO promotes the leucine oxidation and, at the same time, increases the protein degradation rate, leading to negative protein balance and related skeletal muscle atrophy (165). The BCAA undergoes catabolism instead of being incorporated into protein synthesis, providing a carbon substrate for oxidation in the TCA cycle (166). However, evidence concerning heart-specific adaptions in the metabolic process is rare. In an immature swine model, ECMO shifts myocardial metabolic processes toward positive protein balance by preserving BCAA for protein synthesis instead of promoting their oxidation (167). Because ECMO significantly reduces myocardial oxygen consumption, the overall LV leucine oxidation rate is reduced compared with the increase observed in the whole-body-infant study. Besides, pyruvate utilization seems to prevent insulin resistance, further preserving BCAA from oxidation. Simultaneously, ECMO promotes the oxidation of long chain FAs oxidation and inhibits lactate oxidation (168). This metabolic shift during ECMO appears to be explained by rapid upregulation of PDH kinase-4 (PDK4) protein. As demonstrated by Randle et al. (169), PDH is phosphorylated and inhibited by PDK4, thereby reciprocally increasing FAO *via* acyl-CoA dehydrogenases. ECMO also increased the relative flux from lactate to alanine, further supporting the inhibitory effect of PDK4 on PDH (168). In a VA-ECMO immature piglet model, weaning from ECMO circuits induces a sudden increase in cardiac work and myocardial oxygen consumption, which is associated with shifts in substrate utilization and pathways contribution. The exogenous pyruvate is favored vs. those derived from glycolysis, and acetyl-CoA is generated from endogenous substrate, presumably fatty acids and ketones rather than pyruvate decarboxylation (170). In conclusion, in normal hearts, ECMO preserved positive protein balance *via* promoting the FAO and pyruvate oxidation, and successful weaning will not increase the oxidation of amino acids, nor will it adversely affect the rate of protein synthesis.

### VA-ECMO on Mitochondrial Metabolism of Ischemic Hearts

To determine whether VA-ECMO protects the ischemic heart through ameliorating metabolic abnormalities induced by IR, metabolites of neonatal piglets that undergo coronary occlusion for 30 min, followed by ECMO support, have been analyzed (170). The ratio of phosphocreatine to adenosine triphosphate (PCr/ATP) is analyzed as a surrogate for mitochondrial oxidative capacity (171), and it falls when ATP demand nears or exceeds the maximal ATP production rate. Files demonstrated that, compared with the preischemic level, IR significantly decreased the PCr/ATP by > 50%, and ECMO after injury returns the PCr/ATP to near preischemic levels, yet this effect is lost during weaning (172). This suggests that mitochondrial ATP synthesis operates near maximal capacity, as weaning ECMO elevates the energy requirement and disrupts the PCr/ATP. Along with the impaired mitochondrial respiration, IR significantly expanded the intracellular pyruvate pool, and shifted pyruvate from oxidation toward anaerobic glycolysis, indicting by reducing pyruvate decarboxylation (PDC) relative to citric acid cycle (CAC) flux (172). ECMO significantly increased the pyruvate fractional contribution, while weaning followed a similar pattern (172). Besides, the CAC flux is also impaired after ischemia and during reloading. IR did not alter the level of citrate and α-KG but did substantially expand the pools of distal CAC intermediates succinate, fumarate, and malate. The accumulation of succinate is induced by forward flux from α-KG with partial inhibition of SDH within mitochondrial complex II. The high concentrations of succinate might readjust activity of dehydrogenase and produce fumarate and malate at a higher concentration. Alternatively, metabolic analysis suggests that the expansion of oxaloacetate and malate was partially through the anaplerotic entry of pyruvate. As demonstrated previously, IR can promote the carboxylation of anaplerotic pyruvate, which is converted to oxaloacetate by its carboxylase or converted to malate by malic enzyme (173, 174). The metabolic impairments, including the reduced PDC rate and accumulated succinate, also recurred with ECMO weaning, implying that limited OXPHOS due to insufficient acetyl-CoA and disrupted cycling of CAC impaired weaning.

In another adult swine model subjected to LAD occlusion, VA-ECMO was activated followed with 180-min LAD reperfusion, and the hemodynamic parameters were detected. Compared with the group of IR injury, ECMO initiation reduces right atrial RA pressure and LV stroke work but increases the infarct area normalized to the risk area and the infarct area normalized to the total LV area (139). Metabolic data based on unbiased and blinded analysis show that LV unloading by ECMO before reperfusion does not improve the use of myocardial energy substrates, nor can it preserve mitochondrial structure, including cardiolipin content induced by IR injury. Further functional analysis of the mitochondria of infarct zone showed that reperfusion that followed ECMO support also has impaired ETC, including mitochondrial Complexes I, II, and III, and increased oxidative stress with IR. Therefore, despite the fact that ECMO does reduce LV stroke work, it increases the infarct area and fails to protect myocardial mitochondrial and energy metabolism. It should be noted that, except for the hemodynamic effects, the large surface areas of extracorporeal circuits can activate neutrophils, which has already been demonstrated in patients with CS, and the increased circulating neutrophils can mediate reperfusion injury (175). In addition, the data that bypass the left atrium to the femoral artery without an oxygenator can reduce LV stroke work and infarct size also proves this point (133).

In addition to immune alterations, the discrepancies of the two preclinical studies may be attributed to differences between experimental models, such as animal maturity, duration and severity of ischemia, timing of ECMO initiation, flow of ECMO, timing of sampling, etc. Besides, the native cardiac volume and hemodynamic parameters are to be continuously monitored, since they are also correlated with tissue perfusion. As successful weaning of ECMO is based on end-organ function and myocardial recovery, which includes myocardial energetics and substrate utilization, metabolic pathways may be promising therapeutic targets in future clinical practices. Moreover, combined with profound hemodynamic monitoring, metabolites may be used as biomarkers to develop more comprehensive and superior algorithms or models for evaluating myocardial functional recovery.

## Conclusion

This study summarizes substrate use and metabolic alterations during the process of IHD, with an emphasis on IR injury and HF. Although numerous metabolic processes outlined here are ideal drug targets for IR injury, further pieces of research are still needed to gain full insights into the underlying mechanism. Particularly, by unloading the ischemic heart and establishing the oxygen supply-demand balance, MCS is promising in reducing IR injury and improving cardiac function. However, their effects on mitochondrial metabolism and cardiac improvement, especially VA-ECMO, still vary under different conditions. Therefore, more studies are needed to elucidate whether MCS has a beneficial impact on cardiac function and long-term prognosis.

## Author Contributions

MJ wrote the draft of the manuscript and prepared the figures. XX reviewed the manuscript. FC and YW proposed the original idea and reviewed the manuscript. All the authors read and approved the final submitted version of the manuscript.

## Funding

This work was supported by Beijing Municipal Natural Science Foundation (7202189), Project of National Clinical Research Center for Geriatric Disease (NCRCG-PLAGH-2019024), The National Key Research Program of China (2018YFC0116305), The National Natural Science Foundation of China (grant nos. 91939303 and 81820108019), and Major Program of Central Health Committee (2020ZD05).

## Conflict of Interest

The authors declare that the research was conducted in the absence of any commercial or financial relationships that could be construed as a potential conflict of interest.

## Publisher's Note

All claims expressed in this article are solely those of the authors and do not necessarily represent those of their affiliated organizations, or those of the publisher, the editors and the reviewers. Any product that may be evaluated in this article, or claim that may be made by its manufacturer, is not guaranteed or endorsed by the publisher.
